# ACTIVExtend: 24 Months of Alendronate After 18 Months of Abaloparatide or Placebo for Postmenopausal Osteoporosis

**DOI:** 10.1210/jc.2018-00163

**Published:** 2018-05-24

**Authors:** Henry G Bone, Felicia Cosman, Paul D Miller, Gregory C Williams, Gary Hattersley, Ming-yi Hu, Lorraine A Fitzpatrick, Bruce Mitlak, Socrates Papapoulos, René Rizzoli, Robin K Dore, John P Bilezikian, Kenneth G Saag

**Affiliations:** 1 Michigan Bone and Mineral Clinic, P.C., Detroit, Michigan; 2 Division of Metabolism, Endocrinology and Diabetes, University of Michigan, Ann Arbor, Michigan; 3 Department of Clinical Medicine, Columbia University, New York, New York; 4 Clinical Research Center, Helen Hayes Hospital, West Haverstraw, New York; 5 Colorado Center for Bone Research, Lakewood, Colorado; 6 Radius Health, Inc., Waltham, Massachusetts; 7 Center for Bone Quality, Leiden University Medical Center, ZA Leiden, Netherlands; 8 Faculty of Medicine, Geneva University Hospitals, Geneva, Switzerland; 9 David Geffen School of Medicine, University of California at Los Angeles, Los Angeles, California; 10 Division of Endocrinology, Columbia University College of Physicians and Surgeons, New York, New York; 11 Division of Clinical Immunology and Rheumatology, School of Medicine, University of Alabama at Birmingham, Birmingham, Alabama

## Abstract

**Purpose:**

In women with postmenopausal osteoporosis, we investigated the effects of 24 months of treatment with alendronate (ALN) following 18 months of treatment with abaloparatide (ABL) or placebo (PBO).

**Methods:**

Women who completed ABL or PBO treatment in ACTIVE were eligible to receive up to 24 months of ALN. We evaluated the incidence of vertebral and nonvertebral fractures and changes in bone mineral density (BMD) during the entire 43-month period from ACTIVE baseline to the end of ACTIVExtend and for the 24-month extension only.

**Results:**

Five hundred fifty-eight women from ACTIVE’s ABL group and 581 from its PBO group (92% of ABL and PBO completers) were enrolled. During the full 43-month treatment period, 0.9% of evaluable women in the ABL/ALN group experienced a new radiographic vertebral fracture vs 5.6% of women in the PBO/ALN group, an 84% relative risk reduction (RRR, *P* < 0.001). Kaplan–Meier incidence rates for other reported fracture types were significantly lower for ABL/ALN vs PBO/ALN (all *P* < 0.05). Gains in BMD achieved during ACTIVE were further increased during ACTIVExtend. For ACTIVExtend only, RRR for vertebral fractures was 87% with ABL/ALN vs PBO/ALN (*P* = 0.001). Adverse events were similar between groups. A supplemental analysis for regulatory authorities found no hip fractures in the ABL/ALN group vs five in the PBO/ALN group.

**Conclusions:**

Eighteen months of ABL followed by 24 months of ALN reduced the risk of vertebral, nonvertebral, clinical, and major osteoporotic fractures and increased BMD. Sequential ABL followed by ALN appears to be an effective treatment option for postmenopausal women at risk for osteoporosis-related fractures.

## Teaser

In ACTIVExtend, reductions in fracture risk and increases in BMD achieved with 18 months of abaloparatide for postmenopausal osteoporosis were sustained through 24 months of subsequent alendronate.

Anabolic drugs are important therapeutic agents for the treatment of osteoporosis. To date, anabolic drugs approved for the treatment of osteoporosis act via PTH receptor type 1 (PTH1R). Although there are differences between the agents, they also share common characteristics. They stimulate bone formation and resorption and have important effects on bone microstructure, mass, and strength, while transiently increasing the remodeling space (1). Regulatory authorities limit the use of anabolic drugs for postmenopausal osteoporosis to 18 to 24 months. An important characteristic typical of PTH and PTH-related agents is loss of bone mass that occurs soon after they are discontinued when an antiresorptive agent is not subsequently administered (2). Whereas bone anabolic agents increase the volume of bone, leading to an increase in measured bone mineral density (BMD), antiresorptive agents increase BMD mainly by decreasing the remodeling space and increasing the degree of mineralization. For this reason, sequential therapy with alendronate (ALN) following PTH 1–84 was employed in an extension of the phase 2 trial, resulting in further gains in BMD (3). Investigations of sequential and combination therapeutic schemes employing PTH1R-mediated agents and antiresorptives have subsequently been performed (4–6). The overall result has been that such studies, as well as recent studies of romosozumab followed by an antiresorptive (7, 8), have demonstrated cumulative gains in BMD when a drug that promotes bone formation is followed by an antiresorptive. However, until now, an adequate, formal fracture-endpoint trial of sequential therapy with a PTH1R-mediated anabolic agent followed by a potent antiresorptive agent has not been reported. As a result, the prescribing information for such agents has not addressed measures to sustain their beneficial effects.

Abaloparatide (ABL) is a PTH-related peptide analog that increased BMD and reduced fracture risk in postmenopausal women with osteoporosis (9). Fracture risk reduction with ABL at vertebral and nonvertebral sites was rapid and robust. Furthermore, ABL had advantageous effects on BMD in comparison with teriparatide. Based on prior experience with agents employing related mechanisms of action, continuation of therapy with an antiresorptive agent was considered necessary to sustain the effects of ABL. Hence, ACTIVExtend was undertaken to formally assess the longer-term safety and efficacy of extended treatment with ALN following 18 months of ABL or placebo (PBO). The initial results during the first 6 months of ALN in the extension study were previously reported (10). The final results provide new information about the clinical effect of antiresorptive treatment in participants who had been treated with ABL in comparison with those who had been treated with PBO.

## Methods

### ACTIVE description

Inclusion criteria for ACTIVE have been described in detail by Miller *et al.* (9). ACTIVE enrolled 2463 postmenopausal women with osteoporosis, aged 49 to 86 years. Women ≤65 years of age who had radiographic evidence of vertebral fracture at any time or who had a nonvertebral fracture within 5 years were eligible when they also had a BMD T-score of less than −2.5 but greater than −5.0 at the lumbar spine (LS) or femoral neck (FN). Women who were >65 years of age who met these fracture criteria were allowed to enroll when their LS or FN BMD T-score was less than −2.0 but greater than −5.0. Women older than 65 years could also enroll when their LS or FN BMD T-score was less than −3.0 but greater than −5.0, even when they did not meet the fracture criteria. ACTIVE participants were randomized in a 1:1:1 fashion to receive 18 months of treatment with either blinded daily subcutaneous injections of ABL (80 μg) or matching PBO or daily open-label subcutaneous injections of teriparatide (20 μg).

**Figure 1.**
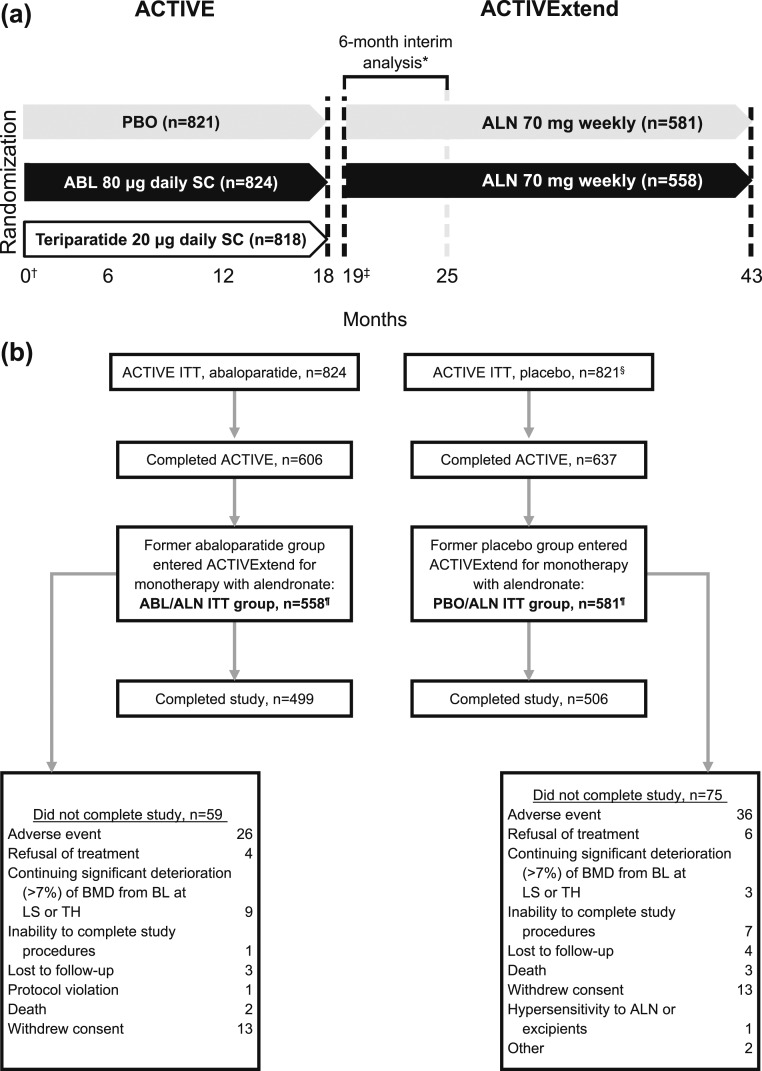
Study design and participant enrollment and disposition. (a) Study design. Participants were treated with ABL, teriparatide, or PBO for 18 mo in ACTIVE. Participants who received ABL or PBO during ACTIVE were eligible for enrollment in ACTIVExtend. A gap in treatment of up to 1 mo (from mo 18 to 19) was allowed for rollover and reconsenting from ACTIVE to ACTIVExtend. Former ABL and former PBO participants began monotherapy with ALN at cumulative mo 19 for up to 24 mo. (b) Participant enrollment and disposition. *As reported by Cosman *et al.* (10). ^†^Baseline for the integrated 43-mo analysis was day 1 of ACTIVE. ^‡^Baseline of the ACTIVExtend analysis was day 1 of ACTIVExtend (cumulative mo 19). ^§^As reported by Miller *et al.* (9). ^¶^A modified intent-to-treat (mITT) population, including those participants with an evaluable postbaseline spine radiograph assessment, was evaluated for incident new vertebral fractures. Fourteen ABL/ALN participants (one because she was not included in the mITT population for ACTIVE and 13 because they did not have a postbaseline spinal radiograph) were not included in the ACTIVExtend mITT, yielding an ABL/ALN mITT of n = 544; 13 PBO/ALN participants (two because they were not included in the ACTIVE mITT and 11 for no postbaseline spinal radiograph) were excluded from the mITT, yielding an mITT n = 568. BL, baseline.

As described by Cosman *et al.* (10), following completion of ACTIVE, there was an interval of ∼1 month during which eligible participants were recruited, gave informed consent, and were enrolled into ACTIVExtend. Thus, there was an off-treatment period of up to 40 days, followed by 24 months on ALN, for a total of 43 months included in the integrated ACTIVE–ACTIVExtend study period [Fig. 1(a)].

### ACTIVExtend inclusion and exclusion criteria

Women who completed visit 9, the end-of-treatment visit for ACTIVE, and who had been randomized to either blinded ABL or blinded PBO were eligible for enrollment into ACTIVExtend when they were appropriate candidates for treatment with ALN, were >80% compliant with study medication during ACTIVE, when not >40 days had passed since their visit 9, and when they had signed a new written informed consent for the extension study. Women were excluded from ACTIVExtend when they had a treatment-related serious adverse event (AE) during ACTIVE, had stopped taking study medication, were noncompliant, or had withdrawn from ACTIVE.

Efficacy and safety were analyzed according to the ACTIVE treatment group assignment (either ABL or PBO); hereafter, the ACTIVExtend treatment groups are designated ABL/ALN and PBO/ALN. Study personnel and participants remained blinded to initial treatment assignment (ABL or PBO) while participants were receiving open-label ALN (70 mg) weekly during the first 6 months of therapy.

The study was conducted in accordance with the ethical principles contained in the Declaration of Helsinki and in compliance with Good Clinical Practice guidelines and all other applicable local regulatory and ethical requirements.

### Primary efficacy endpoint

Efficacy and safety endpoints for the first 6 months of ACTIVExtend have been described in detail (10). The primary efficacy endpoint of the integrated ACTIVE–ACTIVExtend study was the proportion of participants with one or more incidents of new vertebral fracture as determined by the method of Genant *et al.* (11) comparing the ACTIVE baseline spine x-rays with the spine x-rays at the end of ACTIVExtend (*i.e.*, at cumulative month 43). The population evaluable for the primary efficacy endpoint was designated as the modified intent-to-treat (mITT) population.

### Additional efficacy endpoints

Additional efficacy endpoints for the integrated ACTIVE–ACTIVExtend study included time to first incident nonvertebral, clinical, and major osteoporotic fracture from ACTIVE baseline through cumulative months 25, 31, 37, and 43; mean percentage change from ACTIVE baseline at months 25, 31, 37, and 43 in BMD at LS, total hip (TH), and FN; and median percentage change from ACTIVE baseline at months 25, 31, 37, and 43 in serum procollagen type I N-terminal propeptide (s-PINP) and serum carboxyl-terminal cross-linking telopeptides of type I collagen (s-CTX).

### Prespecified exploratory efficacy endpoints in ACTIVExtend using ACTIVExtend baseline

Prespecified exploratory efficacy analyses using the ACTIVExtend baseline (cumulative month 19, the beginning of treatment with ALN) included incidence of new vertebral fracture at months 6 and 24 of ACTIVExtend. Times to first incidents of nonvertebral fracture, clinical fracture, and major osteoporotic fracture were analyzed with the Kaplan–Meier method. Mean absolute changes from ACTIVExtend baseline in BMD at LS, TH, and FN and median percentage changes from ACTIVExtend baseline in s-PINP and s-CTX were analyzed at ACTIVExtend months 6, 12, 18, and 24.

### Safety endpoints

Safety findings reported in the present study are for the 24 months of monotherapy with ALN administered to all participants in ACTIVExtend. These include AEs, vital signs, ECGs, clinical laboratory evaluations (including hematology, serum chemistry, and urinalysis), and ABL antibody assessments. Participants were withdrawn from the study when they had a confirmed >7% decrease from baseline of ACTIVExtend in BMD, treatment-related serious AEs, refusal of treatment, the inability to complete the study procedures, or when they were lost to follow-up. Treatment-emergent AEs were coded using the *Medical Dictionary for Regulatory Activities*, version 17.1.

### Study visits

The ACTIVE follow-up visit, visit 10, ∼1 month after participants’ visit 9, corresponded to day 1 of ACTIVExtend. Vital signs, ECG, clinical laboratory measurements, and bone turnover markers were assessed on day 1 of ACTIVExtend as baseline values for the 24-month ALN treatment period. A total of six clinic visits were scheduled during ACTIVExtend at day 1 and months 3 (cumulative month 22), 6 (cumulative month 25), 12 (cumulative month 31), 18 (cumulative month 37), and 24 (cumulative month 43).

In ACTIVExtend, each participant was assigned to receive 24 months of open-label ALN (70 mg) orally once per week. In addition to ALN, all participants also received supplemental calcium (500 to 1000 mg) and vitamin D (400 to 800 IU) at dosages determined by the investigator. Participants were instructed to take their first dose of ALN within 1 week of their day 1 visit. At ACTIVExtend study month 6, 12, 18, and 24 visits, safety laboratory tests were performed, participants underwent dual-energy x-ray absorptiometry of the hip and spine for measurement of BMD, and fasting blood samples were drawn for bone turnover markers. At the ACTIVExtend month 6 visit, spinal x-rays were performed to diagnose incident vertebral fractures, whereas nonvertebral fractures were determined clinically and confirmed by radiographic reports and/or radiographs. At each visit, participants had a diary review and were questioned about use of concomitant medications and occurrence of AEs. At month 24 of ACTIVExtend, participants had clinical and radiologic fracture assessments, and any AE or clinical laboratory abnormality that was recorded at the month 24 visit was subsequently monitored until it resolved or had become chronic or stable.

### Statistical analysis

For the integrated ACTIVE–ACTIVExtend analyses, the results from the final assessment made before or at the initiation of study drug in ACTIVE were used as baseline. For the exploratory ACTIVExtend 24-month efficacy analyses and the ACTIVExtend safety analyses, baseline measurements are defined as those collected at ACTIVExtend baseline (cumulative month 19, beginning of treatment with ALN).

Fisher’s exact test was used to evaluate between-treatment differences in the incidence of new vertebral fracture, and the log-rank test, the Kaplan–Meier method, and the proportional hazard model were used to assess time to first incident nonvertebral, clinical, and major osteoporotic fracture. The analysis of covariance model with missing data imputation by last observation carried forward was used for BMD at each anatomic site. The mixed-effect repeated measure model was used to assess log ratio of postbaseline over baseline value for bone turnover maker levels. Also reported in this study is an additional analysis of fracture endpoints requested by regulatory authorities to evaluate both the ACTIVExtend intent-to-treat (ITT) population and the full ACTIVE ITT population, including participants who did not continue into ACTIVExtend.

## Results

Of the 1243 women who completed the ABL or PBO arm of ACTIVE, 1139 (92%) were enrolled in ACTIVExtend beginning 20 November 2012. The mean (SD) duration between the last dose of ABL or PBO and the first dose of ALN was 33.8 (8.04) days for ABL/ALN and 33.6 (8.02) days for PBO/ALN treatment groups. A total of 1005 participants completed the 24-month treatment period with ALN monotherapy [Fig. 1(b)]. Participants from the former ABL and former PBO groups who entered ACTIVExtend were well matched for demographic and baseline characteristics and were representative of the ACTIVE population in whole. The characteristics of those patients at ACTIVE baseline and at ACTIVExtend baseline are presented in Table 1. At ACTIVExtend baseline, mean BMD levels and the proportion of participants with vertebral and nonvertebral fractures differed between the two groups, consistent with the prior treatment of ABL vs PBO.

**Table 1.** Demographics and Baseline Characteristics of the ACTIVExtend Population at Baseline of ACTIVE and at the Baseline of ACTIVExtend (Cumulative Mo 19)

	At ACTIVE Baseline		At ACTIVExtend Baseline	
Characteristic	PBO/ALN (n = 581)	ABL/ALN (n = 558)	PBO/ALN (n = 581)	ABL/ALN (n = 558)
Age				
Mean, y (SD)	68.5 (6.3)	68.6 (6.5)	70.1 (6.29)	70.2 (6.54)
<65, n (%)	114 (19.6)	106 (19.0)	86 (14.8)	93 (16.7)
65 to < 75, n (%)	370 (63.7)	351 (62.9)	359 (61.8)	327 (58.6)
≥75, n (%)	97 (16.7)	101 (18.1)	136 (23.4)	138 (24.7)
Race/ethnicity, n (%)				
White	447 (76.9)	433 (77.6)	447 (76.9)	433 (77.6)
Asian	106 (18.2)	101 (18.1)	106 (18.2)	101 (18.1)
Black or African American	18 (3.1)	19 (3.4)	18 (3.1)	19 (3.4)
Other	10 (1.7)	5 (0.9)	10 (1.7)	5 (0.9)
Hispanic or Latino	139 (23.9)	124 (22.2)	139 (23.9)	124 (22.2)
Mean years since menopause (SD)	19.8 (7.9)	20.4 (8.2)	21.3 (7.94)	21.9 (8.23)
Region, n (%)				
North America	7 (1.2)	9 (1.6)	7 (1.2)	9 (1.6)
South America	157 (27.0)	145 (26.0)	157 (27.0)	145 (26.0)
Europe	312 (53.7)	305 (54.7)	312 (53.7)	305 (54.7)
Asia	105 (18.1)	99 (17.7)	105 (18.1)	99 (17.7)
Mean body mass index, kg/m^2^ (SD)	25.0 (3.50)	24.9 (3.49)	25.0 (3.62)	24.9 (3.67)
Prevalent vertebral fracture at baseline, n (%)	132 (22.8)*^a^*	123 (22.0)	140 (24.1)	124 (22.3)
At least one prior*^b^* nonvertebral fracture, n (%)	282 (48.5)	272 (48.7)	293 (50.4)	277 (49.6)
Mean BMD T-score (SD)				
LS	−2.91 (0.82)	−2.88 (0.86)	−2.87 (0.867)	−2.11 (0.997)
TH	−1.91 (0.76)	−1.88 (0.72)	−1.93 (0.758)	−1.63 (0.742)
FN	−2.17 (0.68)	−2.15 (0.62)	−2.20 (0.695)	−1.95 (0.656)

Some data in this table originally appeared in Cosman *et al.* (10).

^a^ Of 580 participants.

^b^ Based on fractures that occurred prior to ACTIVE baseline visit, excluding those of spine, breast bone, knee cap, toes, fingers, skull, and facial bones.

**Figure 2.**
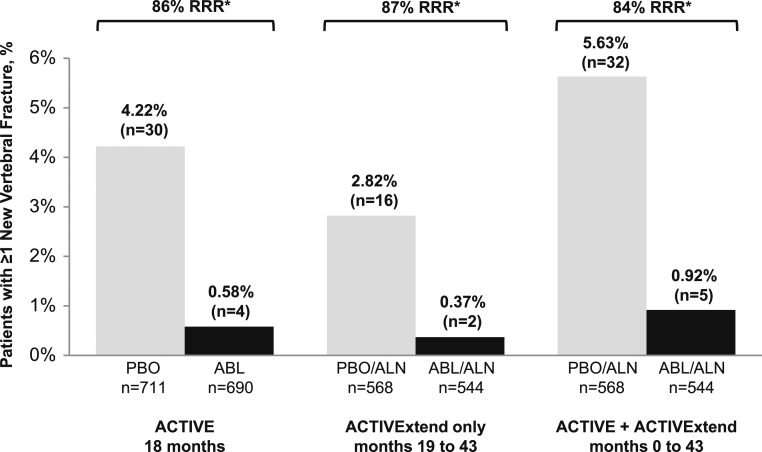
Incidence of new vertebral fractures in ACTIVE, ACTIVExtend only, and ACTIVE plus ACTIVExtend. mITT populations, representing those participants who had baseline and also postbaseline spinal x-rays at the specified time points, were evaluated for vertebral fracture rates. In ACTIVE, treatment with ABL was associated with an 86% RRR for new vertebral fractures compared with PBO. A gap in treatment of up to 1 mo (from mo 18 to 19) was allowed for rollover and reconsenting from ACTIVE to ACTIVExtend. During the ACTIVExtend period only (mo 19 to 43), prior treatment with ABL was associated with an RRR of 87% compared with prior treatment with PBO. For the full ACTIVE/ACTIVExtend study period (mo 0 to 43), treatment with ABL was associated with an 84% RRR compared with PBO. *P ≤ 0.001 for ABL vs PBO and for ABL/ALN vs PBO/ALN. ACTIVE findings were reported by Miller *et al.* (9).

At the end of the full 43-month treatment period of the integrated ACTIVE–ACTIVExtend study, the 86% relative risk reduction (RRR) of new vertebral fractures that had been demonstrated during 18 months of treatment in ACTIVE was effectively sustained. After 18 months of treatment with ABL followed by 24 months of ALN, 0.9% (n = 5) of evaluable women in the ABL/ALN group experienced a new radiographic vertebral fracture, whereas after 18 months of PBO followed by 24 months of treatment with ALN, 5.6% (n = 32) of evaluable women in the PBO/ALN group experienced a new radiographic vertebral fracture, representing an RRR of 84% (*P* < 0.001; Fig. 2).

**Figure 3.**
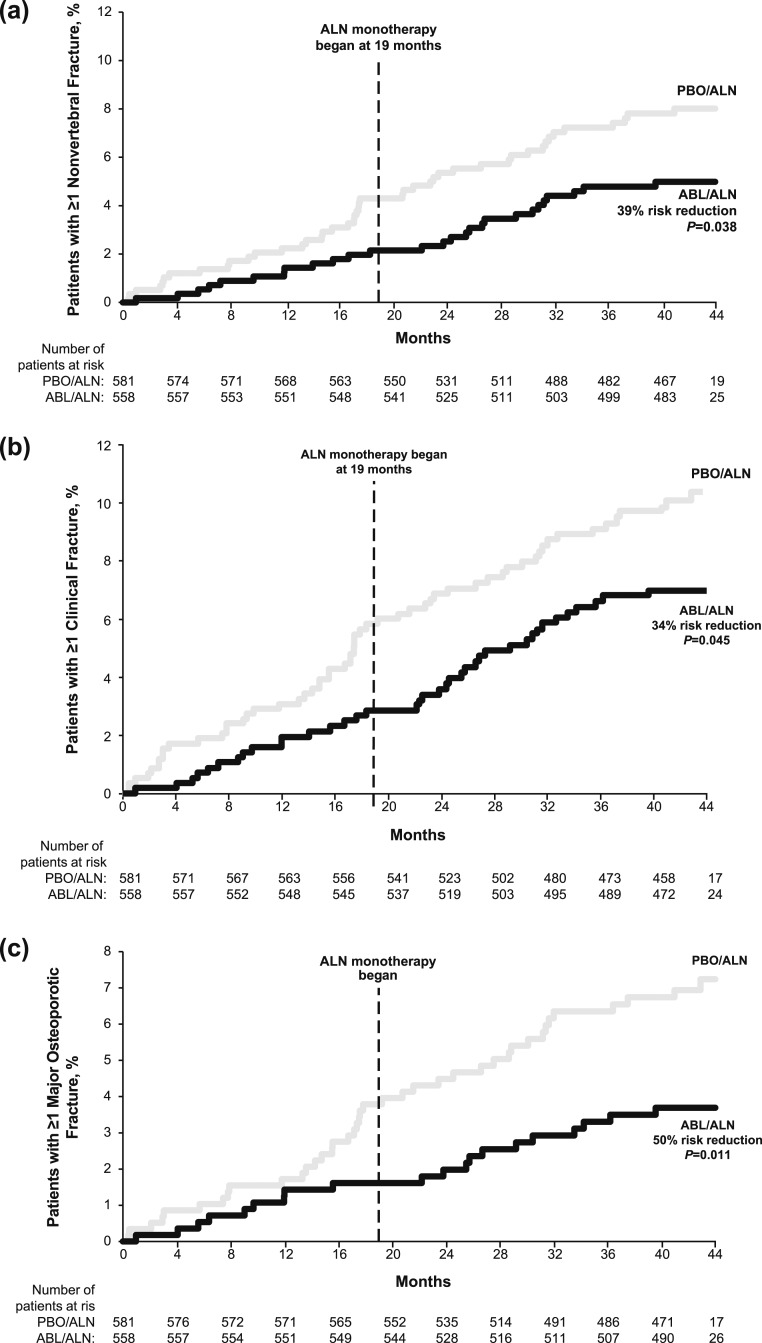
Time-to-event analyses of nonvertebral fractures, clinical fractures, and major osteoporotic fractures from ACTIVE baseline at mo 43. Curves indicate time to the first event. (a) Nonvertebral fractures were defined as fractures excluding those of the spine, sternum, patella, toes, fingers, skull, and face and those with high trauma. (b) Clinical fractures were defined as all fractures that would cause a patient to seek medical care, regardless of the level of trauma, including clinical spine. (c) Major osteoporotic fractures were defined as fractures of the wrist, upper arm, hip, and clinical spine. A gap in treatment of up to 1 mo (from mo 18 to 19) was allowed for rollover and reconsenting from ACTIVE to ACTIVExtend. Risk reduction = (1 − HR) × 100. Data for mo 0 to 18 originally appeared in Miller *et al.* (9).

Kaplan–Meier incidence rates for nonvertebral, clinical, and major osteoporotic fractures for the integrated ACTIVE–ACTIVExtend analysis are summarized in Supplemental Table 1. Incidence rates for all three fracture types were significantly lower in the ABL/ALN group compared with the PBO/ALN group. The separation from PBO observed at month 18 of ACTIVE was sustained at cumulative month 43 (month 24 of ACTIVExtend) for all three fracture types (Fig. 3), with significant risk reductions in the ABL/ALN group of 39%, 34%, and 50% compared with the PBO/ALN group for nonvertebral, clinical, and major osteoporotic fractures, respectively (all *P* < 0.05). Supplemental Table 1 also displays the ACTIVE ITT plus ACTIVExtend ITT analysis, which included participants treated in ACTIVE who did not subsequently enroll in ACTIVExtend. Five participants in the PBO group and no participants in the ABL group had an incident hip fracture during the full 43 months in this combined population (*P* = 0.027).

**Figure 4.**
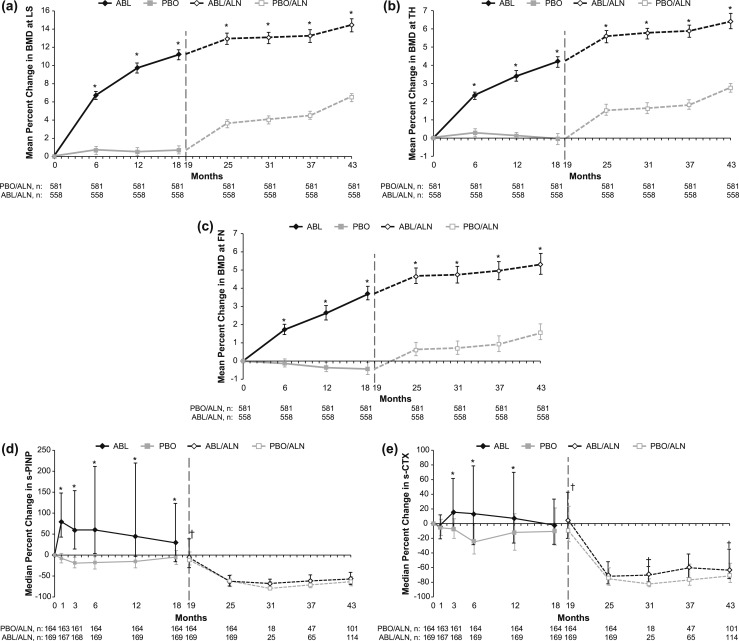
Percentage changes from ACTIVE baseline to end of ACTIVExtend in BMD and in bone turnover markers. (a–c) Mean percentage changes from baseline in BMD at: (a) LS, (b) TH, and (c) FN. Error bars represent 95% CIs. (d and e) Median percentage changes in s-PINP (d) and in s-CTX (e). Error bars represent interquartile ranges. A gap in treatment of up to 1 mo (from mo 18 to 19) was allowed for rollover and reconsenting from ACTIVE to ACTIVExtend. Former PBO and former ABL participants began monotherapy with ALN at mo 19. *P < 0.001 for ABL vs PBO and for ABL/ALN vs PBO/ALN; ^†^*P* < 0.05 for ABL/ALN vs PBO/ALN. Data for mo 0 to 18 originally appeared in Miller *et al.* (9), and data for mo 25 originally appeared in Cosman *et al.* (10).

Percentage changes from baseline in BMD at the LS, TH, and FN for the integrated ACTIVE–ACTIVExtend analysis are shown in Fig. 4. At each anatomic site, gains in BMD realized during ACTIVE with ABL treatment relative to PBO were sustained during 24 months of monotherapy with ALN.

The bone turnover markers s-PINP and s-CTX were evaluated in a subset of participants from each treatment group as shown in Fig. 4. During treatment with ABL in ACTIVE, s-PINP increased to a peak median value of ∼79% greater than baseline values at month 1 before decreasing to ∼30% above baseline values at month 18. During treatment with PBO in ACTIVE, s-PINP decreased from baseline, reaching a nadir of 19% below baseline values at month 3 of ACTIVE. Among participants treated with ABL in ACTIVE, s-CTX increased to a peak of ∼15% greater than baseline at month 3 before decreasing to baseline levels by month 18. Participants in the PBO group in ACTIVE had decreases from baseline in s-CTX, reaching a nadir of 24% lower than baseline values at month 6 of ACTIVE. At month 19, by which time participants had been off ABL-SC and PBO for ∼30 days, s-PINP and s-CTX levels were approaching ACTIVE baseline levels. As expected, during treatment with ALN (months 19 to 43), levels of both s-PINP and s-CTX decreased well below ACTIVE baseline levels by month 25 and remained suppressed through month 43 in both the ABL/ALN and PBO/ALN treatment groups.

### Prespecified exploratory analyses of endpoints from ACTIVExtend baseline to end of study

As described previously, a prespecified exploratory analysis of the 24 months of ALN treatment (from ACTIVExtend baseline to the end of the 24-month treatment period with ALN monotherapy, corresponding with cumulative months 19 to 43) was performed for fracture endpoints. For radiographically determined vertebral compression fractures, from ACTIVExtend baseline to end of study, there was an RRR of 87% (*P* = 0.001; Fig. 2) in the ABL/ALN group [n = 2 (0.37%)] compared with the PBO/ALN group [n = 16 (2.82%)]. Analysis of the incidences of nonvertebral, clinical, and major osteoporotic fractures for the 24-month ACTIVExtend treatment period demonstrated fewer fractures in the ABL/ALN group for all three fracture types, but these differences were not statistically significant compared with the PBO/ALN group: 15 nonvertebral fractures in ABL/ALN vs 20 in PBO/ALN [hazard ratio (HR), 0.76; *P* = 0.422]; 23 clinical fractures in ABL/ALN vs 24 in PBO/ALN (HR, 0.98; *P* = 0.941); 12 major osteoporotic fractures in ABL/ALN vs 17 in PBO/ALN (HR, 0.72; *P* = 0.374; Supplemental Table 1).

During the 24-month ACTIVExtend period, BMD increased at the LS, TH, and FN sites for both groups, as shown in Fig. 4. However, the magnitude of the differences between groups did not increase, but rather decreased slightly: at the start of the extension, BMDs in the former ABL group were significantly higher than in the former PBO group, as previously reported (9). Therefore, percentage changes from the beginning of the extension cannot be compared, and absolute BMD changes are reported. During the 24-month ACTIVExtend period, BMD increased from ACTIVExtend baseline for both groups, but the between-groups differences were small. Mean (SD) absolute increases in BMD from ACTIVExtend baseline to ACTIVExtend month 24 at the LS were 0.0265 (0.0451) and 0.0479 (0.0378) for the ABL/ALN and PBO/ALN groups, respectively (*P* < 0.001). For the TH, the respective gains were 0.0166 (0.0249) and 0.0210 (0.0205) (*P* = 0.001), and for the FN, the gains were 0.0114 (0.0285) and 0.0143 (0.0264) (*P* = 0.073). Median percentage changes in s-PINP at the end of ACTIVExtend were similar in both groups (ABL/ALN, −58.4%; PBO/ALN, −59.2%; *P* = 0.387), as were median percentage changes in s-CTX (ABL/ALN, −64.6%; PBO/ALN, −64.9%; *P* = 0.152).

### Safety and AEs

The overall incidence of AEs, including severe and serious AEs, during the ALN treatment period was similar for both study groups (Table 2) and was consistent with the recognized profile of ALN. The most common treatment-emergent AEs (TEAEs) were arthralgia, upper respiratory infection, and back pain. The incidence of serious TEAEs was 11.8% in the ABL/ALN group and 10.0% in the PBO/ALN group; 5.4% of the ABL/ALN group and 6.2% of the PBO/ALN group had at least one TEAE that led to study drug discontinuation. No cases of atypical femoral fracture or osteonecrosis of the jaw were reported.

**Table 2.** Treatment-Emergent AEs in ACTIVExtend, Cumulative Mo 19 to 43

Summary of TEAEs, ACTIVExtend Safety Population (N = 1133)	PBO/ALN (n = 580), n (%)	ABL/ALN (n = 553), n (%)
≥1 TEAE	466 (80.3)	452 (81.7)
≥1 Severe TEAE	40 (6.9)	38 (6.9)
≥1 Serious TEAE	58 (10.0)	65 (11.8)
≥1 TEAE leading to death	2 (0.3)	0
≥1 TEAE leading to discontinuation	36 (6.2)	30 (5.4)
Most frequently (≥5% in either group) reported TEAEs		
Arthralgia	58 (10.0)	54 (9.8)
Upper respiratory tract infection	51 (8.8)	40 (7.2)
Back pain	34 (5.9)	36 (6.5)
Hypertension	33 (5.7)	27 (4.9)
Pain in extremity	31 (5.3)	23 (4.2)
Osteoarthritis	21 (3.6)	28 (5.1)

## Discussion

The results of ACTIVE and the completed ACTIVExtend trial in postmenopausal women at high risk for fracture demonstrate the effectiveness of a treatment sequence comprised of initial anabolic treatment with ABL for 18 months, followed by continuation of treatment with ALN for up to 2 additional years. The results include favorable effects on fracture risk across anatomic sites and consistent, progressive gains in BMD at the TH, FN, and LS. Although the study was not designed to assess hip fracture risk, an ACTIVE ITT plus ACTIVExtend ITT analysis requested by regulatory authorities demonstrated no hip fractures in the ABL/ALN group vs five in the PBO/ALN arm.

Because the effects of anabolic agents on bone mass are generally reversible if the treatment is discontinued, extension therapy should be considered as a component of any treatment plan with an anabolic agent. Rittmaster *et al.* (3) demonstrated that ALN following PTH 1–84 resulted in not only preservation but also a further increase in BMD. This point has been reinforced by subsequent clinical trials (5, 8, 12), and the approach has recently been reviewed by Cosman *et al.* (13). However, such a treatment sequence has not been tested until now in a fracture-endpoint trial employing a PTH1R-mediated anabolic agent. The efficacy of ABL demonstrated in ACTIVE followed by 24 months of ALN in ACTIVExtend provides evidence to support the use of sequential therapy with 18 months of ABL followed by ALN. This information should be of use to clinicians.

During the extension, both groups received ALN, but the former ABL group had an RRR for new vertebral fractures during the extension of 87%, virtually identical to the large RRR with ABL vs PBO in ACTIVE. This relative reduction in the risk of fracture is all the more impressive in comparison with an active control of known efficacy. It suggests that advantageous effects of ABL on bone strength were maintained on ALN treatment, whereas they were not achieved on ALN alone. Bone resorption, as indicated by s-CTX, increased transiently but was back to baseline by the end of ACTIVE. Thus, we did not see an extra increase in BMD on ALN as we might have expected if the bone resorption rate were still high at the transition. In fact, the increases in BMD on ALN differed little between groups, with a slightly greater gain in the former PBO groups at some anatomic sites. Furthermore, the RRR for nonvertebral, clinical, major osteoporotic, and hip fractures tended toward a more favorable effect of the ABL/ALN sequence. Thus, there appears to be a cumulative benefit that favors the ABL/ALN sequence of treatment over ALN monotherapy introduced at the time of the transition from ABL to ALN.

A sequential treatment strategy using ABL followed by ALN is attractive for several reasons: ABL produces a prompt and consistent reduction in fracture risk, associated with increased BMD at vertebral and hip sites (9), further increasing bone mass by reducing the remodeling space and increasing mineralization. Although anabolic therapies are generally administered for ≤2 years, ALN can be administered for a substantially longer period, with a sustained effect on BMD lasting several years beyond the end of active treatment (14). Thus, an ABL/ALN sequence can provide a rapid and substantial initial benefit as well as a sustained favorable effect, employing a relatively inexpensive medication in the continuation phase.

This study has limitations with respect to its size and duration. It was not powered to demonstrate a significant reduction in nonvertebral fractures during the extension period; studies of greater size and duration would be needed to confirm the favorable trend observed. The only antiresorptive agent evaluated was ALN, but that drug has been extensively evaluated during many years, and it is a widely used antiresorptive agent internationally. Whereas our results are specific to the agents tested, future studies may address the use of other agents or longer term treatment and the use of a similar strategy in other populations.

## Conclusion

Eighteen months of treatment with ABL followed by 24 months of treatment with ALN reduced the risk of vertebral, nonvertebral, clinical, and major osteoporotic fractures and produced large increments in BMD of the LS, TH, and FN. These findings demonstrate that the sequence of ABL followed by ALN can be a highly efficacious treatment option for postmenopausal women who are at risk for osteoporosis-related fractures.

## Supplementary Material

Supplemental Table 1

## Abbreviations:

ABL: abaloparatide
AE: adverse event
ALN: alendronate
BMD: bone mineral density
FN: femoral neck
HR: hazard ratio
ITT: intent-to-treat
LS: lumbar spine
mITT: modified intent-to-treat
PBO: placebo
PTH1R: PTH receptor type 1
RRR: relative risk reduction
s-CTX: serum carboxyl-terminal cross-linking telopeptides of type I collagen
s-PINP: serum procollagen type I N-terminal propeptide
TEAE: treatment-emergent adverse event
TH: total hip
