# Supplementary material for: ACTIVExtend: 24 Months of Alendronate After 18 Months of Abaloparatide or Placebo for Postmenopausal Osteoporosis
Source: J Clin Endocrinol Metab. 2018 May 24;103(8):2949–57. doi: 10.1210/jc.2018-00163 (PMC6097601; doi:10.1210/jc.2018-00163)
Supplement: Supplemental Table 1 [file jc.2018-00163.st1.pdf]

Supplemental Table 1. Kaplan-Meier Rates of Nonvertebral Fracture Endpoints.

	**ACTIVExtend ITT, Cumulative Treatment Months 0 to 43**		**ACTIVExtend Only, Cumulative Treatment Months 19 to 43**		**ACTIVE ITT + ACTIVExtend ITT, Cumulative Treatment Months 0 to 43**	
**Fracture type**	**PBO/ALN, n=581**	**ABL/ALN, n=558**	**PBO/ALN, n=581**	**ABL/ALN, n=558**	**PBO + PBO/ALN, n=821**	**ABL + ABL/ALN, n=824**
**Nonvertebral**						
Participants, n	45	27	20	15	53	33
KM rate, %	8.0	5.0	3.7	2.8	8.4	5.5
HR (95% CI)		0.61 (0.38, 0.98)		0.76 (0.39, 1.49)		0.63 (0.41, 0.98)
P-value		0.038		0.422		0.038
**Clinical**						
Participants, n	58	38	24	23	72	49
KM rate, %	10.4	7.0	4.6	4.3	11.3	8.1
HR (95% CI)		0.66 (0.44, 0.99)		0.98 (0.55, 1.73)		0.69 (0.48, 0.99)
P-value		0.045		0.941		0.045
**Major osteoporotic**						
Participants, n	40	20	17	12	51	21
KM rate, %	7.2	3.7	3.2	2.3	8.2	3.5
HR (95% CI)		0.50 (0.30, 0.86)		0.72 (0.34, 1.50)		0.42 (0.25, 0.70)
P-value		0.011		0.374		<0.001
**Hip**						
Participants, n	3	0	3	0	5	0
KM rate, %	0.6	0	0.6	0	0.8	0
HR (95% CI)		NE		NE		NE
P-value		0.085		0.086		0.027

Abbreviations: **ABL**, abaloparatide; **ALN**, alendronate; **HR**, hazard ratio; **ITT**, intent-to-treat; **KM**, Kaplan-Meier; **NE**, not evaluable; **PBO**, placebo.
